# Marijuana for glaucoma treatment: a recipe for disaster

**DOI:** 10.5935/0004-2749.20210056

**Published:** 2025-02-02

**Authors:** Fábio B. Daga, Izabela Almeida, Tiago S Prata, Augusto Paranhos Jr

**Affiliations:** 1 Brazilian Glaucoma Society; 2 Department of Ophthalmology, Universidade Federal de São Paulo, São Paulo, SP, Brazil

Glaucoma is the leading cause of irreversible blindness worldwide^(1)^. It occurs more frequently in elderly individuals, but it can also develop in young adults, children, and newborns. Glaucoma is characterized by the dysfunction and loss of retinal ganglion cells (RGCs), with corresponding structural changes in the optic nerve, and visual field loss, ultimately leading to blindness^(2)^. Although the mechanism behind RGC damage in glaucoma is not fully understood, intraocular pressure (IOP) reduction decreases the development and progression of the disease^(3)^.

Several drugs can be used to treat glaucoma, such as prostaglandin analogs, beta-blockers, alpha-agonists, carbonic anhydrase inhibitors, and cholinergic agonists. A combination of these drugs is also available for patients who have not achieved adequate IOP control. In addition, laser trabeculoplasty can be used to increase aqueous outflow facility through the trabecular meshwork and reduce IOP in cases of open-angle glaucoma. Conventional incisional procedures, such as trabeculectomy and glaucoma drainage devices, are usually indicated for cases wherein a more pronounced IOP reduction is necessary. More recently, so-called minimally-invasive glaucoma surgeries have been presented, which focus mostly on increasing aqueous humor outflow.

In recent years, the use of alternative therapies have been increasing in Western cultures, especially for the treatment of glaucoma, whether for hypotensive or neuroprotective effects^(4)^. It is estimated that 5% of glaucoma patients use alternative therapies^(5)^. One of these possible alternatives is the use of marijuana. Marijuana remains an illegal substance; however, canabidiol (CBD), one of its non-psychoactive compounds, has recently been approved for restricted medical use in Brazil and other countries. Historically, medical marijuana refers to the use of *Cannabis sativa* and its derivatives, such as CBD and tetrahydrocannabinol (THC), to treat disease or to relieve disease, symptoms^(6)^. The use of medical marijuana dates back 400 years. Despite having several side effects (e.g. chemical dependence, psychiatric disorders, cardiac, hepatobiliary, and gastrointestinal effects), several countries, such as Canada, Czech Republic, Germany, Italy, Holland, and 23 American states, have authorized the use of marijuana for medical purposes.

The possible therapeutic benefits of marijuana have been described for several debilitating medical conditions, such as refractory seizures, neuropathic chronic pain, vomiting and nausea in multiple sclerosis, AIDS, cancer, and glaucoma^(7)^. The marijuana hypotensive effect was first described in 1971. Since then, studies have confirmed its effect through a variety of routes, including inhalation, oral, intravenous, sublingual, and topical (eye drops)^(8)^. The exact mechanism of IOP reduction is unclear, but there is evidence that the hypotensive effect of marijuana on arterial pressure would result in a temporary reduction of IOP^(8)^. Nonetheless, one must consider whether a significant reduction of systemic blood pressure would increase glaucomatous damage. Systemic arterial hypotension has been shown to lead to inadequate blood flow to the optic nerve, which could result in glaucomatous progression^(9)^. Another possible explanation for the reduction of IOP due to marijuana use is related to the ocular cannabinoid receptors, primarily CB1 in the ciliary body, which can decrease the production of aqueous humor when it is activated^(10)^.

Given its transitory hypotensive effect on IOP, a marijuana cigarette would need to be smoked 8 or 10 times per day to achieve a consistent IOP reduction^(11)^. This estimated frequency is based on the fact that IOP reduction induced by marijuana usually lasts for 3-4 hours, with a peak of 60-90 minutes^(11)^. This daily frequency would likely increase the risk of drug dependence. In addition, only 60%-65% of individuals experience a significant IOP reduction of ~25%, which is smaller than the reduction obtained with traditional treatments. Finally, there is evidence that marijuana is associated with progressive tolerance, which may result in a loss of effectiveness over time^(12)^.

With regard to patients’ tolerance to the treatment of glaucoma with marijuana compounds, in a previous study using THC tablets, all patients discontinued its use due to the side effects^(13)^. Given that glaucoma is an incurable disease that should be monitored and treated throughout life, these results suggest that the systemic use of THC is not a reasonable option for continued glaucoma treatment. Moreover, the effectiveness of eye drops is lower than that associated with other routes of administration. This is likely because it is still difficult to achieve adequate intraocular concentrations for hypotensive effects, primarily due to the hydrophobic characteristics of cannabinoids, which hinder corneal absorption. In addition, surface irritation and lacrimation reflex may mitigate drug absorption.

There is an intense worldwide debate regarding marijuana legalization for recreational use. Studies have indicated a world prevalence of marijuana chemical dependency of ~11 million individuals in 1990 and 13 million individuals in 2010^(14)^. Others report that occasional and recreational marijuana use can lead to brain lesions in regions such as the nucleus accumbens^(15)^. For example, in the state of Colorado, in the United States, after the legalization of recreational marijuana use, the number of car accidents doubled, in addition to an increase in emergency room visits, psychiatric disorders, and other medical problems in users compared with nonusers. Furthermore, a systematic review evaluating the efficacy and safety of THC-based drugs for the treatment of several medical conditions (e.g. multiple sclerosis, movement disorders, neurodegenerative diseases, chronic pain, and glaucoma) reported contradictory conclusions, with most studies demonstrating ”unclear” or “potential beneficial” results^(16)^. In contrast, in a recent meta-analysis, CBD, the non-psychoactive compound of marijuana, demonstrated positive clinical effects in the treatment of chronic pain and seizures with low adverse effects^(17-19)^. Scientific observations have supported the use of CBD, when produced in accordance with good manufacturing practices, for restricted medical therapeutic use by national health agencies worldwide (FDA, Food and Drug Administration of USA; EMA, European Medicines Agency; and ANVISA, Brazilian Health Surveillance Agency).

In conclusion, at present, there is no evidence to support the medical use of marijuana or its compounds for the treatment of glaucoma due to its transitory effect, psychoactive nature, and addictive characteristics. In addition, there is a lack of evidence of its possible effects on glaucoma disease progression. Moreover, illegally obtained marijuana can have toxic contaminants and a much lower level of the supposed therapeutic compounds. Traditional therapeutic options offer greater medical benefits and fewer side effects, and thus are still the best alternatives for glaucoma treatment.
